# Hierarchical and nested associations of suicide with marriage, social support, quality of life, and depression among the elderly in rural China: Machine learning of psychological autopsy data

**DOI:** 10.3389/fpsyt.2022.1000026

**Published:** 2022-09-26

**Authors:** Xinguang Chen, Qiqing Mo, Bin Yu, Xinyu Bai, Cunxian Jia, Liang Zhou, Zhenyu Ma

**Affiliations:** ^1^Global Health Institute, Xi’an Jiaotong University, Xi’an, China; ^2^Department of Social Medicine, School of Public Health, Guangxi Medical University, Nanning, China; ^3^Guilin People’s Hospital, Guilin, China; ^4^Department of Epidemiology, Universtiy of Florida, Gaineville, FL, United States; ^5^Department of Biostatistics and Epidemiology, School of Public Health, Wuhan University, Wuhan, China; ^6^People’s Hospital of Guangxi Zhuang Autonomous Region, Nanning, China; ^7^Department of Epidemiology, School of Public Health, Cheeloo Medical College, Shandong University, Jinan, China; ^8^The Affiliated Brain Hospital of Guangzhou Medical University, Guangzhou, China

**Keywords:** suicide, rural Chinese, machine learning, depression, quality of life, social support

## Abstract

**Objectives:**

To identify mechanisms underpinning the complex relationships between influential factors and suicide risk with psychological autopsy data and machine learning method.

**Design:**

A case-control study with suicide deaths selected using two-stage stratified cluster sampling method; and 1:1 age-and-gender matched live controls in the same geographic area.

**Setting:**

Disproportionately high risk of suicide among rural elderly in China.

**Participants:**

A total of 242 subjects died from suicide and 242 matched live controls, 60 years of age and older.

**Measurements:**

Suicide death was determined based on the ICD-10 codes. Influential factors were measured using validated instruments and commonly accepted variables.

**Results:**

Of the total sample, 270 (55.8%) were male with mean age = 74.2 (*SD* = 8.2) years old. Four CART models were used to select influential factors using the criteria: areas under the curve (AUC) ≥ 0.8, sensitivity ≥ 0.8, and specificity ≥ 0.8. Each model included a lead predictor plus 8–10 hierarchically nested factors. Depression was the first to be selected in Model 1 as the lead predictor; After depression was excluded, quality of life (QOL) was selected in Model 2; After depression and QOL were excluded, social support was selected in Model 3. Finally, after all 3 lead factors were excluded, marital status was selected in Model 4. In addition, CART demonstrated the significance of several influential factors that would not be associated with suicide if the data were analyzed using the conventional logistic regression.

**Conclusion:**

Associations between the key factors and suicide death for Chinese rural elderly are not linear and parallel but hierarchically nested that could not be effectively detected using conventional statistical methods. Findings of this study provide new and compelling evidence supporting tailored suicide prevention interventions at the familial, clinical and community levels.

## Introduction

### Increasing suicide among senior residents in rural China

Increased rate risk of suicide among seniors has been reported in some developed countries (1–4). The increasing suicide among senior residents in rural China has becoming a significant issue. The unevenly paced development has resulted in growing financial inequality, leading to extra suicide risk for vulnerable subgroups of population (5). Senior residents in rural China represent one of such subgroups. These seniors are vulnerable due to many life-threatening events, including family separation, poor health in general, physical illness and mental health issues in particular (6–9). Economic growth in China has attracted many young and educated farmers from rural areas to urban areas, leaving the elderly with young children at homes (6). Family separation may exert significant pressure on these left-behind seniors, leading to depression and suicide (6). Furthermore, people in old ages often suffer from chronic diseases with poor health status, exacerbating the risk for suicide (5, 8).

In addition to factors related to QOL, family separation, mental health and diseases, old adults who remain in rural homes may experience progressive deterioration in income (6, 8). Despite that most farmer workers in urban areas can find a job, make money and send money back home, not all rural migrants are successful; and for those who are successful, not all of them can have extra money to send home to support their parents and children. Furthermore, it is not uncommon that many farmer workers will not get paid till the end of a year, which also prevents them from sending money back home timely to support their parents and children.

In addition to shortage of income, many old adults experience difficulties in obtaining social support for problem solving, such as access to healthcare, and labor shortage for farming and childcaring during special seasons (6, 8). Furthermore, it is not uncommon for one spouse of a married couple who remains in rural home to deal with unstable marriage and divorce after the other one moved to urban areas to make money. For these old adults, unstable marriage may become the deepest and most significant risk for suicide (6).

### The need for new analytics to advance suicide research

Much has been documented regarding influential factors of suicide for old adults in rural China. Typical examples include depression and other mental health issues (6), poor QOL (9, 10), lack of social support (11, 12), lack of education, low income (13, 14), unstable marital status (15, 16) and gender (17, 18). However, when these factors are analyzed together in a multivariate model, many are no longer statistically significant, suggesting the lack of independent effect of individual factors. Taking depression and poor QOL as an example, when analyzed separately, these two factors are significantly associated with suicide in various populations (19–21), including seniors in rural China (22, 23). However, when these two factors are analyzed in one model, QOL is no longer significant (24). This finding does not indicate the lack of influence of QOL but a more complex relation between QOL, depression and suicide that cannot be detected using the conventional methods.

The need for different analytical approaches is further supported by recent system reviews and meta-analyses of suicide studies conducted in different countries and regions in the past 50 + years. Multivariate analytical approaches such as logistic regression, mediation and moderation analysis, and structural equation modeling have been widely used in published studies, but the established models often performed poorly in predicting suicide with estimated AUC (area under the curve) rarely greater than 0.60 and sensitivity and specificity rarely greater than 70% (25, 26). The relationship between multiple risk factors and suicide is much more complex than independent and linear. To extract convincing evidence from data supporting suicide prevention and control, new and more innovative analytical approaches are needed.

### Machine learning as an alternative

Machine learning (ML) provides a promising alternative to the conventional multivariate regression approach. One typical ML method is the classification and regression tree (CART) (27–29). Different from the conventional approaches of multivariate linear regression, multinomial logistic regression, multivariate logistic regression, multivariate ordinal logistic regression, ML is data-driving that can disentangle complex relationships between many predictors and an outcome based on the significance of individual predictors. Therefore, ML can also avoid *variable mask effect* as in multivariate regression in which a proximal variable (i.e., depression) can block the effect of a distal variable (i.e., QOL) on suicide. Additionally, variables detected through decision trees in CART are linked hierarchically, thus providing further information on potentially nested hierarchical relationships among key predictors. Lastly, ML makes it possible to test different risk scenarios by purposefully excluding some key variables. ML algorithms such as CART/decision tree, random forest, and deep learning have already shown its superiority to the conventional approaches in predicting suicidal behaviors (30–32) and death (33, 34) in other populations.

### The purpose of this study

In this study, we examined factors related to suicide for old adults in rural China using CART, taking advantages of the psychological autopsy data collected by a case-control project. The goal is to provide new data beyond linear relationships to advance our understanding of suicide and better inform evidence-based precision interventions.

## Materials and methods

### Research design, participants and data

Data were from a suicide study project targeting rural residents 60 years of age and older. Procedures for sampling and data collection were detailed elsewhere (6). Briefly, three provinces in China were randomly selected based on the gross domestic production (GDP); and 12 counties were randomly selected from the three provinces using the level of economic development. Suicide cases were identified based on death certificates from the County Death Registries using the ICD-10 criteria. Subjects who died by suicide during the study period from February 2014 to September 2015 were included.

For each suicide case, a 1:1 match was used to select living controls. The criteria used to select controls were: living in the same neighborhood, the same gender, and within a similar age range (± 3 years old).

Data were collected using classic psychological autopsy technique (35, 36). Interviewers were faculty members and graduate students from three universities in the sampled provinces that co-hosted the project. All interviewers received a 10-day training on psychological autopsy method. Data for cases were collected from informants using questionnaire. Two informants per subject were recruited, including family members, neighbors, friends, and other important persons of the participants.

Institutional Review Board (IRB)‘s approval for data collection was obtained from the Shandong University, the Central South University and Guangxi Medical University in China; and IRB approval for data analysis was obtained from University of Florida in the US.

### Variables and measurement

A total of 15 predictor variables were included for this study based on the suicide literature.

#### Demographic and socioeconomic factors (6 variables)

Age (*in year*), gender (*male/female*), marital status (*stable/unstable*), educational attainment (*less than primary, primary, more than primary*), employment (*employed/not*), and personal income (*100 RMB Yuan/year*).

#### Depression

This variable was assessed using the Geriatric Depression Scale (GDS) (37). GDS is a 30-item scale that measures depressive symptoms in the week prior to suicide (for cases) or prior to the interview (for controls). Cronbach alpha was 0.94 from our data, total scores (range, 0–30) were computed such that higher scores indicated more depressive symptoms.

#### Quality of life

This variable was measured using the Quality of Life Scale (38), a 6-item scale tapping the information regarding physical, mental, financial and social wellbeing. A typical item was “How do you rate your physical health status in the past month?,” varying from 1 (*very poor*) to 5(*excellent*). Cronbach alpha = 0.78 from correlation analysis and GFI = 0.97 from confirmative factor analysis. Total scores were computed (range: 6–30) with higher scores indicated better QOL.

#### Social support

This variable was measured using the Duke Social Support Index (DSSI) (39). DSSI uses 23 items to evaluate social interaction, subjective and instrumental social support. DSSI scores significantly predicted suicide in a previous study (40). The Cronbach alpha = 0.89 in the current study.

#### Variables for living arrangement

Two variables were: (1) If left behind (yes/no): Subjects were coded as “yes” if all adult children left rural homes for urban areas for at least 10 months during the past year and did not visit home more than twice; otherwise, “no.” (2) Living alone (yes/no): This variable was measured using the question: “With whom you are currently living?” Subjects responded “No one” were coded as “yes”; otherwise, “no.”

#### Perceived burdens to family

Two variables were: (1) Economic burden (yes/no) was measured using the question: “Have you ever thought that your diseases added an economic burden to your family?” (2) Physical and mental burden (yes/no) was measured using the question: “Have you ever thought that your diseases brought a physical and/or mental burden to your family?” The two questions were measured using the Likert scale varying from 1 (*not at all*) to 4 (*very much*). Subjects with response “4” were coded as “yes”; otherwise, “no.”

#### Health status in general (poor/not poor)

This variable was assessed using the question: “How do you rate your own health in general?” Answer option varied from 1 (*very bad*) to 5 (*very good*). Subjects with response of 4 or less were coded as “poor”; otherwise, “not poor.” (2) *Having a chronic disease* (yes/no): This variable was assessed using the question: “Have you ever been diagnosed with a severe or chronic disease?” with answer options of 1 (*yes*) and 0 (*no*).

#### Family suicide history (yes/no)

This variable was assessed using the question: “Has any member in your family ever attempted suicide or committed suicide?” with answer options of 1 (*yes*) and 0 (*no*).

### Statistical analysis

Descriptive (mean, standard deviation, proportion, and rate) and bivariate (student *t*-test and chi-square test) analyses were used to describe the study sample and to compare the difference in variables between the suicide cases and living controls. CART was used for machine learning to detect predictors using a new procedure we invented. The new procedure, termed as the stepwise exclusion CART modeling, started with the CART to construct the first prediction model by including all 15 predictors and from the 15 to detect the first lead influential factor.

The modeling procedure was repeated to detect the second lead factor among the remaining 14 by excluding the first leader factor. The exclusion CART modeling procedure repeated until a CART model could not be further improved according to the criteria. As a conventional rule (27–29), sensitivity (≥ 0.8), specificity (≥ 0.8 and area under the curve (AUC ≥ 0.8) were used as criteria for data-model fit assessment.

With our stepwise exclusion approach, a total of four lead factors were detected using four CART models. PROC HPSPLIT was used for CART modeling (27) and for each model, AUC, importance and rank of selected variables were estimated. In all modeling analyses, the sample was partitioned into 65% for training and 35% for validation (29).

Comparison with logistic regression. The same variables included in a CART model were analyzed using a conditional logistic regression for case-controlled data. Odds ratio (*OR*) and 95% confidence interval (*CI*) were estimated. Statistical inference was made at *p* < 0.05 level (95% *CI* excluding 1.00).

Data processing and statistical analyses were completed using the commercial software SAS V. 9.14 (SAS Institute, Cary, NC, USA).

## Results

### Study sample and comparability between suicide cases and living controls

Results in Table 1 indicate that the case and control groups were similar in most demographic and socioeconomic variables except two: marital status and employment. Student *t*-test indicated significant differences between cases and controls for GDS scores, DSSI scores, and QOL scores (*p* < 0.01 for all).

**TABLE 1:** Differences in predictors between cases and controls.

Variable	Suicide cases	Living controls	Total
**Sample size, *N* (%)**	242 (50.00)	242 (50.00)	484 (100.00)
Age (year), mean (SD)	74.43 (8.22)	74.05 (8.16)	74.24 (8.19)
**Gender, *n* (%)**			
Male	135 (55.79)	135 (55.79)	270 (55.79)
Female	107 (44.21)	107 (44.21)	214 (44.21)
Marital status, *n* (%)**			
Stable	122 (50.41)	170 (70.25)	292 (60.33)
Unstable	120 (49.59)	72 (29.75)	192 (39.67)
**Educational attainment, *n* (%)**			
Less than primary	111 (45.87)	96 (39.67)	207 (42.77)
Primary	105 (43.39)	116 (47.93)	221 (45.66)
More than primary	26 (10.74)	30 (12.40)	56 (11.57)
Employment, *n* (%)*			
Yes	40 (16.53)	59 (24.38)	99 (20.45)
No	202 (83.47)	183 (75.62)	385 (79.55)
Annual income (100 yuan), mean (SD)	31.68 (58.80)	43.76 (83.98)	37.72 (72.67)
**Living arrangement**			
Being left behind, *n* (%)*	41 (16.94)	25 (10.33)	66 (13.64)
Living alone, *n* (%)**	64 (26.45)	35 (14.46)	99 (20.45)
**Perceived burdens to family**			
Economic, *n* (%)*	146 (60.33)	123 (50.83)	269 (55.58)
Physical/mental, *n* (%)*	134 (55.37)	110 (45.45)	244 (50.41)
**Health status**			
Poor general health, *n* (%)**	200 (82.64)	164 (67.77)	364 (75.21)
Chronic disease, *n* (%)**	202 (83.47)	161 (66.53)	363 (75.00)
Family suicide history, *n* (%)*	62 (25.62)	37 (15.29)	99 (20.45)
GDS score, mean (SD)**	21.41 (5.95)	9.22 (6.42)	15.31 (8.68)
QOL score, mean (SD)**	15.40 (3.11)	19.50 (3.11)	17.45 (3.70)
DSSI score, mean (SD)**	22.88 (5.98)	27.47 (6.82)	25.18 (6.81)

QOL, Quality of Life scale; GDS, Geriatric Depression Scale; DSSI, Duke Social Support Index. *p < 0.05; **p < 0.01.

### Classification and regression tree models and suicide predictors

Table 2 summarizes results of the four CART models with the stepwise exclusion procedure. Estimated AUCs for the training sample varied from 0.96 for Model 1 to 0.82 for Model 4, greater than 0.8, the criterion for this study. Model 1 used 10 of all 15 factors to predict suicide with GDS score (importance = 11.17), DSSI score (importance = 3.22), and QOL score (importance = 2.80) as the three most influential factors. After exclusion of GDS, Model 2 predicted suicide using another 10 factors from the remaining 14, and with QOL score, DSSI score and income as the three most influential factors. Results for all four models are detailed in Table 2.

**TABLE 2:** Predictors from the four CART modeling analyses.

Variables	Model 1		Model 2		Model 3		Model 4	
	Importance	Rank	Importance	Rank	Importance	Rank	Importance	Rank
GDS score	11.17	1	Excluded	n/a	Excluded	n/a	Excluded	n/a
QOL score	2.80	3	8.98	1	Excluded	n/a	Excluded	n/a
DSSI score	3.22	2	4.02	2	6.32	1	Excluded	n/a
Income	1.60	8	3.29	3	1.19	11	3.20	2
Marriage	2.38	4	1.80	8	2.79	4	3.15	3
Education	1.66	7	1.85	7	1.66	10	1.58	10
Poor general health	1.46	9	2.09	4	3.20	2	2.92	5
Family hist.	1.93	5	1.11	10	n/s	n/a	1.95	9
Chronic diseases	1.06	10	n/s	n/a	2.53	5	3.11	4
Employment	n/s	n/a	1.91	6	2.36	7	2.87	6
Physical/mental burden	n/s	n/a	1.46	9	2.45	6	3.24	1
Economic burden	n/s	n/a	n/s	n/a	3.12	3	2.20	7
Living alone	1.72	6	n/s	n/a	2.28	8	1.35	11
Left behind	n/s	n/a	n/s	n/a	1.70	9	2.17	8
Gender	n/s	n/a	1.98	5	n/s	n/a	n/s	n/a
**Performance**								
Training set (0.65)								
AUC	0.96		0.91		0.91		0.82	
Sensitivity	0.95		0.83		0.85		0.71	
Specificity	0.91		0.84		0.84		0.84	
Test set (0.35)								
AUC	0.76		0.74		0.65		0.64	
Sensitivity	0.75		0.68		0.68		0.53	
Specificity	0.80		0.70		0.61		0.74	

Model 1: All 15 variables were included; Model 2: GDS was removed; Model 3: GDS and QOL were removed; Model 4: GDS, QOL, and DSSI were removed. Importance: changes in residual sum of square by adding a variable. GDS, Geriatric Depression Scale; QOL, Quality of Life scale; DSSI, Duke Social Support Index. AUC, Area under curve. n/s: not selected and n/a: not applicable.

### Four typical binary trees

**FIGURE 1:**
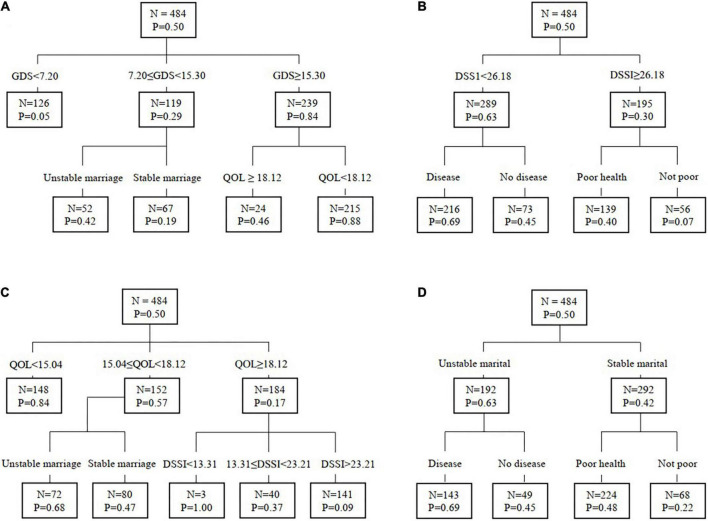
**(A–D)** Four samples of binary decision trees from CART modeling analysis. GDS, geriatric depression scale score; QOL, quality of life scale score; DSSI, duke social support index score; P, proportion of suicide cases.

Figure 1 presents four binary trees corresponding to the four CART models in Table 2. Figure 1A presents the tree from Model 1, in which the total 484 subjects were first divided into three groups with GDS scores = 7.20 and 15.30 as cutoffs. The proportion of suicide cases, from high to low was 84% (*OR* = 16.80) for those with GDS ≥ 15.30, 29% (*OR* = 5.80) for those with 15.30 < GDS ≤ 7.20, and 5% (*OR* = 1.00) for those with GDS < 7.20. The 239 participants with GDS ≥ 15.30 were further divided into two groups with QOL score = 18.12 as the cutoff. The proportion of suicide cases was 88% (*OR* = 1.91) for those with lower QOL relative to 46% for those with higher QOL. Likewise, the 119 participants with 7.20 ≤ GDS < 15.30 were further divided into two groups by marital status with 42% (*OR* = 2.21) of suicide cases for those with unstable marriage relative to 19% for those with stable marriage.

Figure 1B shows a typical tree of Model 2 with GDS excluded. The total sample was first divided into three groups with QOL scores of 15.04 and 18.12 as cutoff points. The proportion of suicide cases from high to low was 84% (*OR* = 4.94) for those with QOL score < 15.04, 57% (*OR* = 3.35) for those with 15.04 ≤ QOL score < 18.12, and 17% (*OR* = 1.00) for those with QOL score ≥ 18.12. The 152 participants with 15.04 ≤ QOL score < 18.12 were further divided into two groups by marital status with 68% (*OR* = 1.45) of suicide cases for those with unstable marriage, and 47% for those with stable marriage.

The remaining 184 subjects with QOL score ≥ 18.12 were divided into three groups with DSSI scores of 13.31 and 23.21 as cutoff points. The proportion of suicide cases from high to low was 100% (*OR* = 11.11) for those with DSSI score < 13.31, 37% (*OR* = 4.11) for those with 13.31 ≤ DSSI score < 23.21, and 9% (*OR* = 1.00) for those with DSSI score ≥ 23.21.

Figure 1C depicts a typical tree of Model 3. In this tree without GDS and QOL, all 484 subjects were first divided into two groups with DSSI = 26.18 as the cutoff; 63% suicide cases were in the lower DSSI-score group and 30% in the higher DSSI-score group (*OR* = 2.10). The 289 subjects in the lower DSSI group were also divided into two groups by chronic diseases with 69% of suicide cases for those with a chronic disease and 45% for those without (*OR* = 1.53). The remaining 185 subjects in the higher DSSI group were also divided into two groups by general health status with higher proportion of suicide cases in the group with a poor health status (40 vs. 7%, *OR* = 5.71).

Figure 1D illustrates a typical tree of Model 4 after exclusion of GDS, QOL and DSSI. In this model, the 484 subjects were first divided into two group by marital status with higher proportion of suicide cases in the group with unstable marriage relative to the stable marriage group (63 vs. 42%, *OR* = 1.50). Subjects with an unstable marriage (*n* = 192) were divided again into two groups by chronic diseases with higher proportion of suicide cases in the group with chronic disease than the group without (69 vs. 44%, *OR* = 1.57). Subjects with a stable marriage (*n* = 292) were also divided into two groups by general health status with higher proportion of suicide cases in the group with poor general health (48 vs. 22%, *OR* = 2.18).

### Results from conditional logistic regression

Results in Table 3 shows that Logistic Regression Model 1 found two from the 15 factors that were significantly associated with suicide: GDS scores with *OR* [95% *CI*] = 1.28 [1.17, 1.41] and employment *OR* = 3.04 [1.07, 8.63]. Logistic Regression Model 2 detected four significant factors: QOL scores with *OR* = 0.66 [0.57, 0.75], employment status with *OR* = 2.09 [1.02, 4.26], marital status with *OR* = 2.37 [1.05, 5.35], and DSSI score with *OR* = 0.91 [0.86, 0.96]. Results from Model 3 were similar to those from Model 2 with perceived physical/mental burden to family and having a chronic disease added. Four significant factors detected by Model 4 were marital status, perceived physical/mental burden, and having a chronic disease.

**TABLE 3:** Odds ratio (95% *CI*) from conditional multivariate logistic regression.

Variable	Model 1	Model 2	Model 3	Model 4
**Demographic**				
Age (in years)	1.13 [0.94, 1.37]	1.09 [0.93, 1.27]	1.09 [0.95, 1.25]	1.12 [1.00, 1.26]
Gender (male = 1)	–	–	–	–
Marriage (unstable = 1)	2.55 [0.91, 7.16]	2.37 [1.05, 5.35]	3.07 [1.55, 6.07]	3.03 [1.63, 5.65]
**Socioeconomic status**				
Education	0.76 [0.32, 1.79]	0.85 [0.49, 1.47]	0.66 [0.40, 1.07]	0.72 [0.47, 1.09]
Employment (yes/no)	3.04 [1.07, 8.63]	2.09 [1.02, 4.26]	1.79 [1.01, 3.15]	1.54 [0.93, 2.54]
Income (100 RMB)	1.00 [1.00, 1.00]	1.00 [1.00, 1.00]	1.00 [1.00, 1.00]	1.00 [1.00, 1.00]
**Living arrangement**				
Being left behind	1.08 [0.24, 4.80]	0.78 [0.27, 2.24]	0.85 [0.37, 1.96]	1.36 [0.63, 2.91]
Living alone	0.91 [0.27, 3.08]	1.13 [0.45, 2.87]	1.03 [0.49, 2.18]	1.06 [0.53, 2.13]
**Perceived burdens to family**				
Economic	1.29 [0.32, 5.11]	1.33 [0.49, 3.57]	1.50 [0.69, 3.27]	1.36 [0.67, 2.74]
Physical/mental	2.38 [0.54, 10.62]	1.82 [0.62, 5.31]	2.71 [1.19, 6.20]	2.71 [1.28, 5.73]
**Health status**				
Chronic disease	2.63 [0.69, 10.00]	2.29 [0.87, 6.00]	6.64 [2.84, 15.49]	6.17 [2.94, 12.94]
Poor general health	0.39 [0.11, 1.36]	0.51 [0.21, 1.22]	1.15 [0.60, 2.20]	1.30 [0.73, 2.34]
Family suicide history	2.13 [0.64, 7.07]	1.78 [0.81, 3.91]	1.62 [0.86, 3.03]	1.59 [0.90, 2.81]
GDS score	1.28 [1.17, 1.41]	–	–	–
QOL score	0.91 [0.77, 1.08]	0.66 [0.57, 0.75]	–	–
DSSI score	0.98 [0.91, 1.05]	0.91 [0.86, 0.96]	0.87 [0.83, 0.91]	–

Model 1: All 15 variables were included; Model 2: GDS was removed; Model 3: GDS and QOL were removed; Model 4: GDS, QOL, and DSSI were removed.

## Discussion

In this article, we reported our research on suicide risk factors among old adults in rural China with a case-control design. Psychological autopsy data were analyzed using CART, an ML method with a stepwise exclusion procedure. Capitalizing on the case-control design with no misclassification in the outcome variable, our analysis has successfully demonstrated the first time that several key risk factors reported by different studies such as depression, QOL, social support and marital status do not affect suicide risk independently but through a hierarchically nested mechanism. This study also detected four different suicide risk scenarios each of which consisting of hierarchically nested risk factors. To our knowledge, this is the first ML suicide risk study of the elderly in rural China. Study findings add new data to the literature on suicidology and provide compelling evidence supporting tailored precision interventions. It is worth noting that the CART and logistic regression are two different analytical methods, each with its own advantages and limitations. The CART is used to enhance our analytical capability, not to replace logistic regression and other methods.

### Depression as the lead predictor

According to results from CART modeling, depression represents the most proximal risk factor for suicide with high accuracy in predicting suicide. Of all 10 variables, depression ranks the first, 3.47 times stronger than the second one (social support), and 3.99 times stronger than the third (QOL). These findings confirm the importance of these factors in affecting suicide among old adults in rural China (5, 21).

CART modeling provides additional information regarding the influence of depression. First, as GDS scores increased, the *OR* for suicide increased from 1.00 with low GDS score, to 5.80 with moderate DGS score, and further to 16.80 with high GDS scores, suggesting a dose-response relation. Secondly, suicide risk for highly depressed subjects can be altered by QOL, suggesting an interaction. Likewise, marital status can alter suicide risk for moderately depressed subjects with an unstable marriage. Lastly, the hierarchical relationship from marital status and QOL to depression, further to suicide risk suggests a complex moderated mediation mechanism.

### Importance of quality of life

Our stepwise CART modeling indicates that QOL represents a suicide risk above depression, forming a 2 = level risk factor structure. After depression was removed, QOL becomes the lead factor. In this scenario, the second and third most important factors are social support and personal income. In addition, in this setting employment, perceived burdens to family, and gender are included; but chronic disease excluded. Different from depression, QOL and suicide showed a reverse dose-response relationship. Furthermore, for subjects with moderate QOL, suicide risk is higher for those with an unstable marriage; while for subjects with high QOL, risk is higher for those lacking social support.

QOL has been associated with suicide among the elderly in China (9, 41) and other countries (42). The current study is the first to delineate a dose-response relationship between the two. Furthermore, although low QOL is a suicide risk, moderate and high QOL can also increase suicide risk for those with an unstable marriage or lack of social support. Likewise, the hierarchical relationship from marital status to social support, further to QOL suggests a potential moderated mediation mechanism underpinning the suicide risk.

### Role of marital status and social support

Our stepwise exclusion CART analysis also found social support and marital status the level-3 influential factor after the removal of depression and QOL. In this hypothetic setting, lack of social support was the lead predictor. Consistent with other studies in China (6, 8, 21) and other countries (21, 43), this study finding confirms the influence of social support in suicidology; what new from our analysis include (1) for subjects with lower social support, their suicide risk can be further increased if also suffering from a chronic disease; (2) for subjects with higher social support, the suicide risk can increase for those with poor general health.

Likewise, in a suicide scenario with no impact of depression, QOL and social support, marital status would be a lead factor. In this condition, subjects with an unstable marriage would be at high suicide risk relative to those with a stable marriage (6, 16, 24). Furthermore, the risk could be exacerbated by chronic diseases for subjects with unstable marriage; and by poor general health for those with stable marriage. The relationship between marital status and suicide among young adults in rural China was reported (44); this study adds new data for the elderlies.

### Implications for suicide prevention and control

Suicide prevention is a global challenge (45, 46). Findings of this study provide new and compelling data supporting suicide prevention interventions among the elderly in rural China. At the individual level, psychiatrists and psychologists can develop new and tailored existent therapies, counseling and other cares based on the hierarchically nested influential factors for different suicide scenarios. For example, for seniors with mental health problems, treating depression remains the first-line intervention. However, for those with moderate depression, unstable marriage should be considered in counseling while for those with more severe depression, QOL should be considered. In addition, attention should be paid to chronic disease and general health status for those with unstable marriage and/or lack of social support even they are not depressed and having good QOL.

The influence of QOL, social support and marital status suggests the importance of community participation and public health policies in suicide prevention for rural seniors with no obvious mental health problems. For those who are left behind and/or live alone, with poor health, and/or having a chronic disease, their suicide risk will increase if lack of QOL and social support or live with an unstable marriage. Therefore, emphasizing community participation to enhance QOL and social support would work for risk reduction. Suicide risk can be further reduced by promoting stable marriage and provision of care for poor health and chronic diseases.

### Machine learning method to advance suicide research

Big data and ML open a new approach toward data analysis for research (47, 48). In this study, we demonstrate several advantages of CART, an ML method recently gaining popularity in suicide research (27–29). In addition to finding risk factors, this method can predict suicide with high accuracy, provide information about potential underlying relationships among predictors, including mediation and moderated mediation mechanisms. Therefore, application of ML in data analysis will allow researchers to systemize knowledge on risk factors, and generate new data better informing interventions.

In addition, we have explored other advantages of ML by incorporating a stepwise exclusion procedure to assess different hypothetic suicide-risk scenarios. ML is a data-driving approach, adding the stepwise exclusion procedure further strengthens the method for researchers to test new theories.

### Limitations and future research

Data used for this study were derived from informants of the subjects, caution is needed in interpreting the findings, despite strengths of the psychological autopsy technique. The interview was held 2–6 months after suicide death, recall bias was thus likely. In addition, sample size can be further increased for subgroup analysis, given the robust result from of our study. Lastly, even though we tested 15 variables, not all factors are included, such as personality trait (49), adverse childhood experience and trauma (50). We will continue our research to examine these other factors in future studies.

## Data availability statement

The raw data supporting the conclusions of this article will be made available by the authors, without undue reservation.

## Ethics statement

The study was approved by the institutional review boards of Shandong University, Central South University, and Guangxi Medical University. The patients/participants provided their written informed consent to participate in this study.

## Author contributions

XC proposed the study, guided and participated in data analysis, and drafted and revised the manuscript. QM participated in conceiving the study, data analysis, and manuscript development. BY participated in data analysis and manuscript development. XB participated in revised the manuscript. ZM, CJ, and LZ conducted the psychological autopsy study and reviewed and commented the manuscript for improvement. All authors contributed to the article and approved the submitted version.
